# Nanoparticle-based delivery of harmine: A comprehensive study on synthesis, characterization, anticancer activity, angiogenesis and toxicity Evaluation

**DOI:** 10.1016/j.heliyon.2024.e31678

**Published:** 2024-05-21

**Authors:** Faezeh Mohammadi, Negar Ghaleh navi, Ehsan Karimi, Masoud Homayouni-Tabrizi, Ehsan Oskoueian

**Affiliations:** a Department of Biology, Mashhad Branch, Islamic Azad University, Mashhad, Iran; b Industrial and Mineral Research Center, Arka Industrial Cluster, Mashhad, Iran

**Keywords:** Nanocarriers, Nanomedicine, Drug delivery, Drug discovery, Targeted delivery, Nanotechnology

## Abstract

The effective treatment of cancer presents numerous challenges, including drug resistance and the risk of detrimental effects on normal tissues. Harmine, a beta-carboline alkaloid, has demonstrated diverse biological properties. This study aimed to synthesize and characterize harmine encapsulated in polylactic-*co*-glycolic acid (PLGA) nanoparticles (Ha-PLGA-NPs) to investigate their potential as agents against cancer and angiogenesis. The synthesized Ha-PLGA-NPs were thoroughly characterized, exhibiting a connected rod-shaped crystal which some retaining the spherical shape of nanoparticles with an average size of 302.96 nm. Furthermore, the nanoparticles demonstrated a dispersion index of 0.23 and a surface charge of −16.51 mV. In vitro cytotoxicity assays conducted on the breast cancer cell line (MCF-7) revealed that Ha-PLGA-NPs possessed significant cytotoxic properties, with an observed IC_50_ value of 87.74 μg/mL. Notably, no substantial cytotoxicity was observed in human foreskin fibroblasts, indicating a favorable selectivity towards cancer cells. Evaluation of the anti-angiogenic activity of Ha-PLGA-NPs demonstrated a concentration-dependent inhibition of angiogenesis. Mechanistic investigations indicated that the observed inhibition was mediated through the regulation of key genes involved in angiogenesis, including caspase 3, caspase 9, VEGF, and VEGF-R. In vivo studies involving dietary administration of Ha-PLGA-NPs in mice revealed improvements in weight gain, feed intake, liver enzyme levels, and redox potential. These findings underscore the potential of Ha-PLGA-NPs as a promising therapeutic agent for cancer treatment. The observed effects are attributed to their ability to induce programmed cell death and inhibit angiogenesis, thus offering a multifaceted approach to combat cancer.

## 1 Introduction

Cancer is a multifaceted and complex disease that is characterized by the uncontrolled proliferation of abnormal cells in the body. These cells, known as cancer cells, deviate from the normal cellular behavior and acquire the ability to evade the body's natural mechanisms of growth regulation and suppression. The pathogenesis of cancer is a complex and dynamic process that involves genetic, epigenetic, and environmental factors. As such, the diagnosis, treatment, and management of cancer require specialized knowledge and expertise. Effective cancer care involves a multidisciplinary approach that integrates the latest scientific advances with personalized patient-centered care [1,2]. Breast cancer is the most common cancer diagnosed in women, accounting for more than 1 in 10 new cancer diagnoses each year. It is the second most common cause of death from cancer among women in the world. It constantly evolves silently without any specific symptoms. Most patients discover their disease during their routine screening. Others may present with an accidently discovered breast lump, change of breast shape or size, or nipple discharge [3,4]. Most chemotherapies have a lot of severe side effects that can damage bone marrow and weaken the immune system, creating an open window for opportunistic infections, and even if the patient survives, because of the damage that has been done, cancer could happen again [5]. Therefore, designing new efficient, and potent drugs with less toxicity and side effects is vital [6].

The plant phytoconstituents such as phenolic, flavonoid, saponin, and alkaloids have been displayed to be valuable, impressive candidates and can be applied for remedies of various illnesses for instance cancer, diabetes, cardiovascular, and Alzheimer's diseases [7,8]. One of these active compounds is harmine, a beta-carboline alkaloid that possesses core indole with a pyridine ring structure and is mainly found in the seed of *Peganum harmal*. previous studies manifested that harmine exhibits significant antitumor activities in vitro and in vivo, including inhibiting proliferation, migration, and invasion, promoting apoptosis, and preventing tumorigenesis [9,10]. Despite its potential benefits in cancer treatment, harmine has some limitations like low absorption, less bioavailability, and might not even be able to reach its target effectively due to oxidation [10,11]. For this purpose, the coherent and well-organized nanocarrier may be a promising approach to enhance controlling release and improve its biological potential [12].

Nanotechnology has overcome the challenging obstacles and limitations of natural bioactive compounds against cancer and various diseases treatment by preparing precise delivery and molecular imaging possibilities [13,14]. In previous research, it was demonstrated that silver nanoparticles (AgNPs) were synthesized from leaf polysaccharide (PS) extracted from *Acalypha indica*. The results showed that PS-AgNPs had a significant ability to scavenge free radicals (with an IC_50_ of 112.91 μg/mL) and also reduced the viability of prostate cancer cells with an IC_50_ value of 101.43 μg/mL [15]. In a series of studies, the green synthesis of silver nanoparticles (AgNPs) using an aqueous leaf extract of *Plumeria alba* was conducted. These studies aimed to determine the antimicrobial potential of the synthesized AgNPs against various pathogenic bacteria and their anticancer activity against glioblastoma MG cancer lines. The results revealed that the synthesized AgNPs exhibited remarkable antimicrobial activity against selected human pathogenic microbes and effective anticancer activity with an IC_50_ value of 9.77 μg/mL [16]. The functionalized stable nanocarriers provide efficient and bio-accessible cancer-selective cytotoxicity in several types of tumors [17]. One of the new nano-delivery systems that have been great attention and applied to enhance natural phytochemicals absorption and develop their solubility is Poly (lactic-*co*-glycolic acid) or PLGA [18].

PLGA is considered to be quite biocompatible, eco-friendly, and less reactive in the body. The key point regarding PLGA biocompatibility is where the polymer is implanted or placed in the body [19,20]. The body could have different immune responses depending on where the polymer is placed. For example, in drug delivery systems, PLGA implants with high surface area and low volume of injection can increase one's chance of immune response as the polymers degrade in the body [21]. We herein aim to design and characterize the PLGA-harmine nanoparticle to develop the delivery of harmine in breast cancer and survey its antiangiogenic potential. Meanwhile, the cytotoxicity activity in regulating the cellular redox state of PLGA-harmine nanoparticles was evaluated in Balb-C mice.

## 2 Material and method

### 2.1 Materials

From Sigma-Aldrich, such products including Harmine (with purity over 95 % and CAS no. of 442-51-3), PLGA, polyvinyl alcohol (PVA), EDC, and NHS were obtained from Merck company, dichloromethane (DCM), 3- (4, 5-dimethylthiazol-2-yl)-2, (MTT) or 5-diphenyltetrazolium bromide, and (DMSO) or dimethyl sulfoxide were purchased from Thermo Fisher Scientific, Uk. The cell lines were provided by the Cell Bank of Ferdowsi University of Mashhad, Iran.

### 2.2 Synthesis and characterization of harmine-loaded PLGA (Ha-PLGA-NPs)

To load the harmine in PLGA NPs using the single emulsion solvent technique W/O, PLGA got solvated in DCM. After the completing organic phase solution with harmine, many different amounts of PVA in two phases were added to the formula. In the first step, 4 mL PVA (2 %) was added to the solution and sonicated to become an emulsion and then in the second step, 10 mL of PVA (0.1 %) was added and incubated on a stirrer for about 2 h. The sample was centrifuged for 20 min at 13000 rpm when the solvent was completely evaporated. The supernatant was collected to check the rate of harmine encapsulation capability [22,23].

To justify harmine-PLGA physiochemical properties, particle size, PDI, and surface charge in triplicate at 25+ and 0.5 and 175^o^C by discharging DLS on zeta sizer (Malvern Instruments, United Kingdom) was controlled. By using the Scanning Electron Microscope (SEM) imaging method (Carl Zeiss, Germany) the size and morphology were measured. The Fourier Transform Infrared Spectrometer (FTIR) was used to discover the functional groups and molecular structures of (Ha-PLGA-NPs).

### 2.3 In vitro anticancer evaluation

The impact of Ha-PLGA-NPs on normal cell lines HFF (Human Foreskin Fibroblasts) and MCF-7 (breast cancer cell line) was surveyed using MTT assay (4,5-3-dimethylthiazolyl) (2, 2-5-diphenyltetrazolium bromide). In short, in every well of a 96-well plate, 10 μl cells per cell line (5000 cells each well) were added and to gain suitable cell growth and cell adhesion, it was incubated for 24 h at 37^o^C and with 5 % CO_2_. Then, the MTT reagent was put in the wells and incubated in the darkness for 2 h. MTT reagent was collected from every well and DMSO (dimethyl sulfoxide) was added to the wells [24]. Finally, spectrophotometry at 570 was used on the plate to measure the absorbance [25].

The following equation shows the percentage of cell viability:

(Absorbance Control – Absorbance treatment)/Absorbance Control × 100 %

### 2.4 Microscopic examination using AO/PI staining

This technique is utilized to analyze alterations in the nucleus of cells and identify the form of cell demise. Essentially, this approach enables the differentiation between living cells (green color), cells undergoing primary apoptosis (yellow to pale orange), secondary apoptosis (bright orange), and necrosis (red). Initially, the cells were cultured in a 6-well plate and subsequently treated with varying concentrations of Ha-PLGA-NPs for 24 h. Following this, the cells were detached using trypsin, centrifuged, and stained with AO/PI dyes in equal amounts (1 μL/mL PBS) before being observed under a fluorescent microscope.

### 2.5 Chick chorioallantois membrane (CAM) assay

To investigate the Ha-PLGA-NPs angiogenic properties, 48 fertilized chicken eggs were sterilized by alcohol and transferred into an incubator (38^o^C; 60 % (v/v) humidity). A tiny hole with a sterilized needle was made for convenient embryo placement. A small window was made on every egg and through that window, Ha-PLGA-NPs at 0, 0.25, 0.5, 1, and 2 mg/mL concentrations were injected into the chorioallantoic sac. By PBS (0.1 M, pH 7.2) thalidomide (1000 μM), the positive and negative control eggs were injected separately. The eggs were incubated in the incubator and the window in the eggshell was sealed with wax before the incubation. After 12 days of incubation, the eggs were opened and the chorioallantoic membrane vasculatures by a microscope equipped with a digital camera (Canon, Japan) were photographed [26]. The software Photoshop CS2 (Image J) was used to identify the angiogenesis, the vessel grade types, and neovascular points.

### 2.6 Apoptotic and anti-angiogenesis gene profiling using Real-Time PCR

The total RNA of the MCF-7 cells was extracted using a Norgen kit to study the apoptosis genes (caspase 3 and caspase 9), after 48 treatments of MCF-7 cells with IC_50_. By using the nanodrop method the amount of extracted RNA was evaluated and mentioned as a template for complementary DNA synthesis. A reaction combination of cDNA, specific primer, SYBR Green, and distilled water with a final volume of 20 μL was prepared and analyzed under a specific temperature-time program in Biorad-CFX96 Real-Time PCR device. As an internal control for normalizing the level of target gene expression, the GAPDH gene was used [27]. As mentioned in Table 1, gene expression of angiogenesis genes like VEGF and VEGFR were notified in the same way with special primers.

**Table 1:** qPCR primer sequences.

**Genes**	**Forward (5′- 3′)**	**Reverse (5′-3′)**	**Organism**
Caspase 3	ctggactgtggcattgagac	acaaagcgactggatgaacc	Homo sapien
Caspase 9	cctcctaaaggacctagaggaaggc	caaggccaggagaggcactggggag	Homo sapien
GAPDH	ccggatcgaccactacctgggcaac	gttccccacgtactggcccaggacca	Homo sapien
VEGF	aaaggccggtacaaaccacc	ttcccccttcttttccgctg	Gallus gallus
VEGF-R	caagtggctaccagtcaggg	ccactggtttcggcctagag	Gallus gallus
GAPDH	ggtggccatcaatgatccct	gcccatttgatgttgctggg	Gallus gallus

### 2.7 Toxicity assay

To assess the toxicity of Ha-PLGA-NPs, 15 female Balb/c mice (at 4 weeks of age) were bought from the laboratory of the animal research center at Razi Vaccine and Serum Research Institute (Mashhad, Iran). The adaptation process between mice and the lab environment took 1 week. Then they were separated into 3 groups randomly including T1 (Control), T2 (50 mg/kg/BW Ha-PLGA-NPs), and T3 (100 mg/kg/BW Ha-PLGA-NPs). 5 mice were put into each cage (30, 15, 15 cm) in a standard climate room (22 + 2^0^C, humidity 50 % + 5 %, 12/12 light/dark cycle) and had access to tap water and food. At the end of 35 days, mice were anesthetized and blood samples were straightly gained from the abdominal aorta because of measuring blood tests analysis using blood auto-analyzer (Hitachi 902, Japan). In 10 % formalin for at least 48 h, tissues like the liver, kidney, intestine, and spleen were isolated and for morphological and histological examination of all tissues, hematoxylin-eosin (H & E) staining was applied. The confirmation of the mice trial in this study was granted by the ethical committee of the Azad University of Mashhad and the laws, norms, and regulations dealing with international animal ethics.

### 2.8 Antioxidant gene expression in mice liver

Briefly, mice livers were smashed and RNA extraction was prepared with an RNeasy Mini kit (Qiagen, Hilden, Germany). After that, Quantitate Reverse Transcription Kit (Qiagen, Hilden, Germany) was used to gain cDNA libraries, and primer sequences (Table 2) aimed for the SOD and house-keeping (GAPDH) genes were designed as well. SYBR Green PCR Master Mix (Qiagen, Hilden, Germany) was used to perform a comparative Real-Time PCR and the amplification of designated genes was under the following program: 95^o^C in 5 min (1X), 95^o^C for 20sec., 55^o^C for 20 s, and 72^o^C for 25 s(35X). The normalization for gene expression to GAPDH was selected as a reference and after, normalized to respective gene expression in the control group.

**Table 2:** The primers were applied in the i*n vivo* experiment.

Gene	Forward (5 ′→3′)	Reverse (5 ′→3′)
SOD	gagacctgggcaatgtgact	gtttactgcgcaatcccaat
GAPDH	gacttcaacagcaactcccac	tccaccaccctgttgctgta
GAPDH: Glyceraldehyde 3-phosphate dehydrogenase; SOD: Superoxide dismutase		

## 3 Results and discussion

### 3.1 Ha-PLGA-NPs physicochemical characterization

**Fig. 1:**
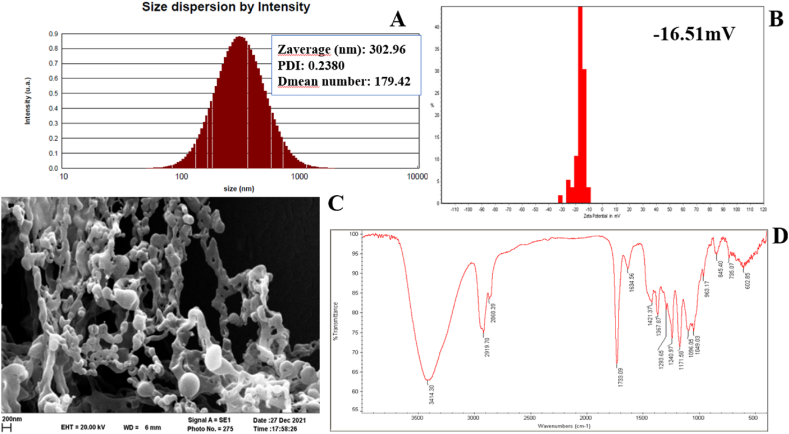
(A) DLS, (B) zeta potential, (C) SEM micrograph, and (D) FTIR spectra results of Ha-PLGA-NPs.

The physicochemical characterization of Ha-PLGA-NPs is essential for understanding their properties and evaluating their potential as drug delivery systems. In this study, several characterization techniques were employed to investigate the size, stability, morphology, and chemical composition of the nanoparticles. In Fig. 1 A, and B, it is shown that Ha-PLGA-NPs have an average size of 302.96 nm, a dispersion index of 0.23, and a surface charge of −16.51 mV.

Based on these results, the size range is within the desirable range for drug delivery applications, as it allows for efficient cellular internalization and distribution. The dispersion index of 0.23 suggests a low level of aggregation or clustering, indicating that the nanoparticles are well-dispersed and stable in the chosen suspension medium. This is a favorable characteristic as it ensures uniformity in drug loading and release. The negative surface charge of Ha-PLGA-NPs suggests the presence of functional groups on the nanoparticle surface, which can contribute to stability by providing electrostatic repulsion between particles. Further, the zeta potential value of −16.51 mV for the Ha-PLGA-NPs revealed that, it possesses incipient instability in the colloidal dispersions. The uniform dispersion and negative surface charge collectively indicate that Ha-PLGA-NPs possess good colloidal stability, minimizing the risk of aggregation or precipitation during storage and administration. The SEM analysis of Ha-PLGA-NPs revealed a connected rod-shaped crystal which some retaining the spherical shape of nanoparticles (Fig. 1C). This shape is advantageous for drug delivery, as it promotes better interactions with cells and tissues. The uniform size distribution further confirms the reproducibility and control achieved in the synthesis process, which is crucial for consistent drug delivery performance.

According to the FT-IR results, the presence of a broad absorption band around 3414 cm^−1^ indicates the presence of the N–H group, which confirms the incorporation of harmine into the nanocomposite structure. The peaks observed around 2919 cm^−1^ are assigned to the aliphatic chain (-CH_3_) of PLGA, indicating the successful encapsulation of harmine within the PLGA matrix. The presence of a band around 1634 cm^−1^ corresponds to the (C=N) bond in the pyridine ring of harmine, confirming the presence of harmine in the nanoparticles. Additionally, the stretching vibrations of the carbonyl (C=O) and -C-O groups appearing around 1733 cm^−1^ and 1171 cm^−1^, respectively, further support the presence of PLGA in the nanoparticles.

Overall, the physicochemical characterization of Ha-PLGA-NPs provides valuable insights into their properties. The nanoparticles exhibit a suitable size, stable dispersion, and spherical morphology. The negative surface charge and uniform distribution suggest good colloidal stability. The FT-IR analysis confirms the successful encapsulation of harmine within the PLGA matrix. These findings collectively contribute to the understanding of Ha-PLGA-NPs as potential drug delivery systems, paving the way for further investigations and applications in the field of cancer therapy [28,29].

### 3.2 Anticancer potential of Ha-PLGA-NPs

**Fig. 2:**
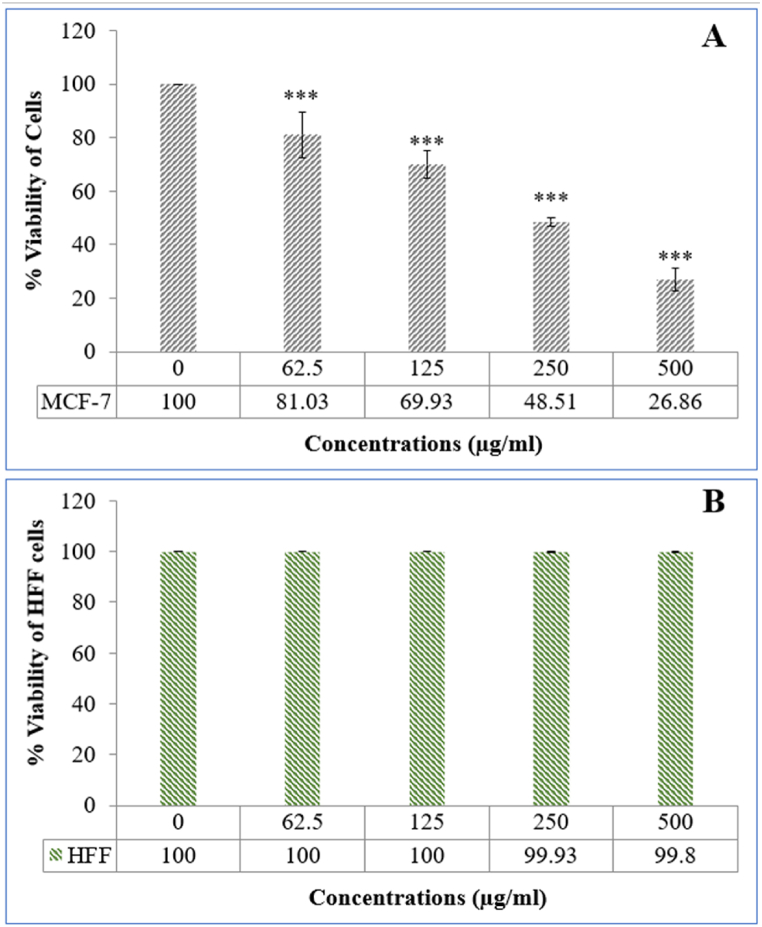
The anticancer impact of Ha-PLGA-NPs on (A) breast cancer cell line (MCF-7) and (B) normal cell (HFF). All values represent the mean ± standard deviation from three independent experiments.

In Fig. 2A, it is evident that the MCF7 breast cancer cell line indicated a significant reduction in cell survival when exposed to various doses of Ha-PLGA-NPs. The IC_50_ value of the Ha-PLGA-NPs was determined to be 87.74 μg/mL. In contrast, the normal cell bioavailability (HFF) was at 99 %, indicating that the nanoparticle had no inhibitory effect (Fig. 2B). Other studies have also shown that when crocetin is encapsulated in PLGA, it has a superior anti-proliferative effect on the MCF7 cancer cell line and a significant reduction in the IC_50_ of crocetin in a dose-dependent manner (IC_50_ = 84.73 μM) [30]. These findings are consistent with several similar studies that have investigated the anticancer potential of PLGA-based nanoparticles. For instance, Smith et al. [31] reported a significant decrease in cell viability of prostate cancer cells upon treatment with PLGA-encapsulated paclitaxel nanoparticles. Similarly, Zhang et al. [32] demonstrated the effective inhibition of lung cancer cell growth using PLGA-based nanoparticles loaded with a specific chemotherapeutic agent. These studies collectively support the notion that PLGA nanoparticles can serve as efficient carriers for delivering anticancer agents and inducing cytotoxic effects in various cancer cell lines.

### 3.3 AO/PI staining analysis of Ha-PLGA-NPs

**Fig. 3:**
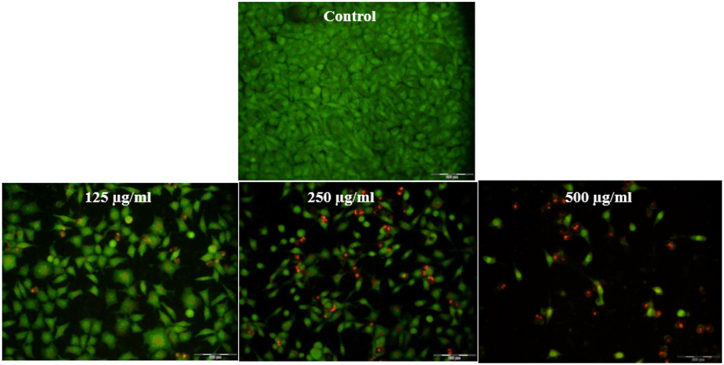
AO/PI staining, which shows an increase in apoptosis in cells treated with different concentrations of nanoparticles compared to untreated cells.

Fig. 3 illustrates that in untreated cells, all cells exhibit a green color, signifying their viability. With the rise in nanoparticle concentration, the proportion of green cells diminishes while the number of yellow and orange cells rises, suggesting the disruption of cell membranes and the penetration of PI dye.

### 3.4 Anti-angiogenic properties of Ha-PLGA-NPs

**Fig. 4:**
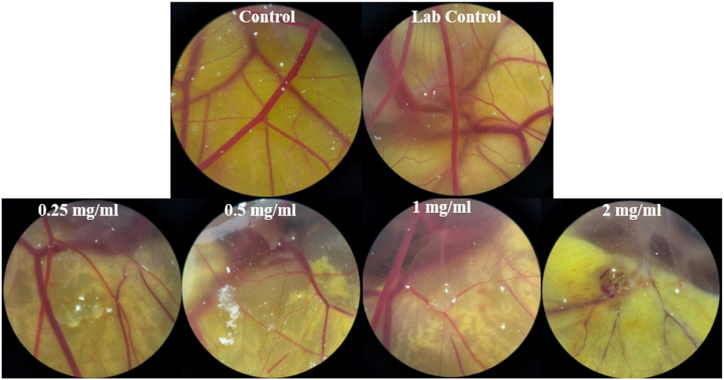
The different concentrations of Ha-PLGA-NPs impact the angiogenesis in the chick embryo chorioallantoic membrane (CAM) assay. The negative and positive control (Lab control) eggs were injected with PBS (0.1 M, pH 7.2) and thalidomide (1000 μM). Cells were visualized at 40X magnification.

**Fig. 5:**
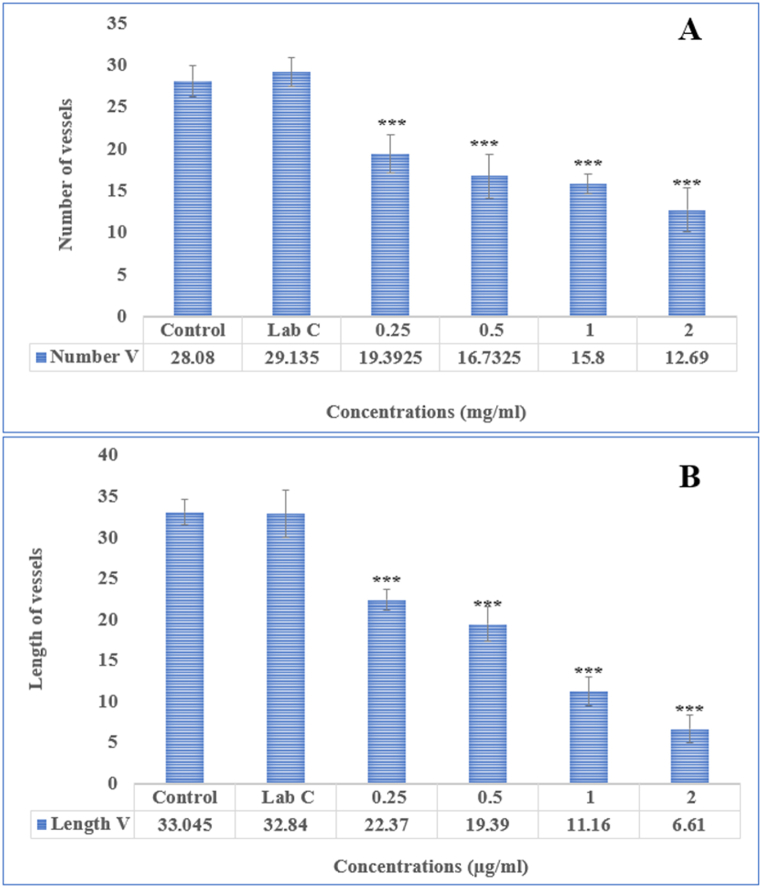
The (A) Number and (B) length of blood vessels treated by different concentrations of Ha-PLGA-NPs compared to the control group (**P < 0.01 and ***P < 0.001).

**Fig. 6:**
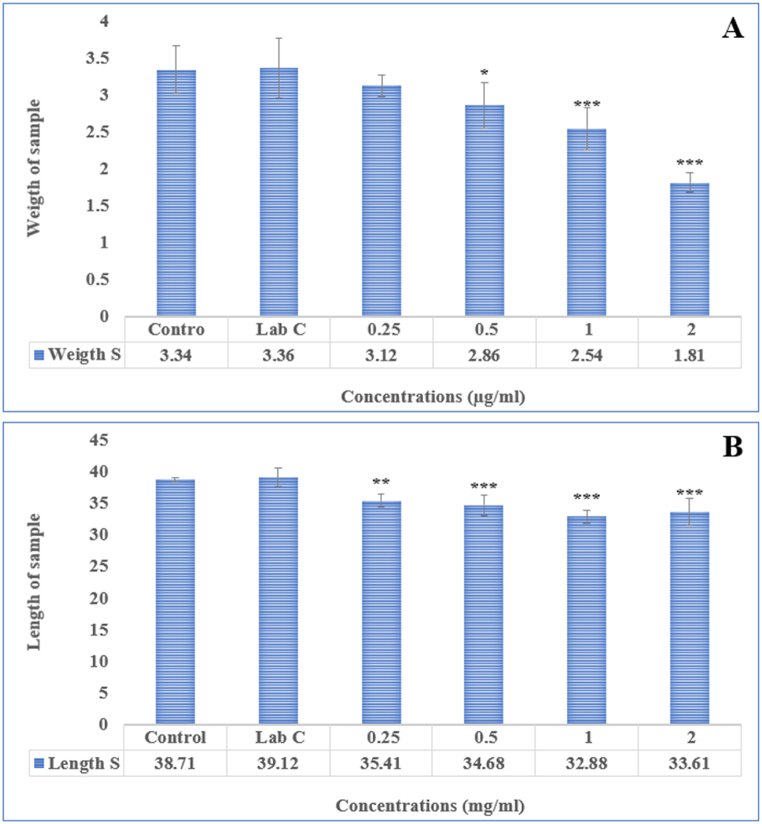
The (A) Weight and (B) length of the sample treated with different concentrations of Ha-PLGA-NPs compared to the control group (**P < 0.01 and ***P < 0.001).

To test the effectiveness of Ha-PLGA-NPs in preventing angiogenesis, a chick chorioallantoic membrane (CAM) assay was conducted and the results are shown in Fig. 4. The findings indicate that the synthesized nanoparticles could significantly inhibit in vivo angiogenesis in a concentration-dependent manner (p < 0.05). Additionally, varying concentrations of Ha-PLGA-NPs were found to inhibit the length and number of blood vessels (Fig. 5 A and B). Furthermore, different concentrations of Ha-PLGA-NPs significantly reduced the weight and length compared to the control (Fig. 6 A and B). Previous research has shown that harmine exhibits anticancer activity through various mechanisms, including induction of apoptosis, cell proliferation inhibition, and angiogenesis modulation [33]. For instance, Liu et al. [34] reported the anti-angiogenic properties of harmine in breast cancer by suppressing VEGF expression and inhibiting angiogenesis. Angiogenesis plays a crucial role in tumor growth and metastasis by supplying nutrients and oxygen to the developing tumor. Therefore, targeting angiogenesis is an essential strategy in cancer therapy [35,36].

### 3.5 Apoptosis and anti-angiogenic related genes profiling

**Fig. 7:**
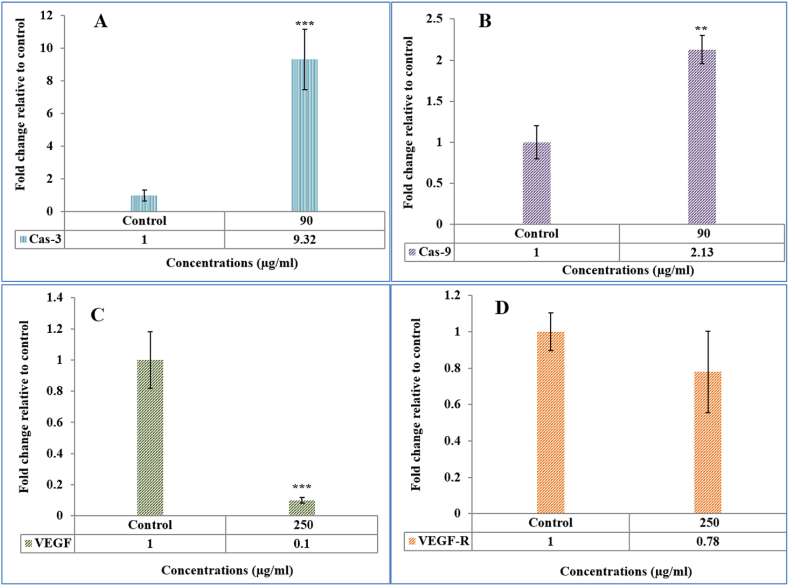
The Ha-PLGA-NPs impact on gene profiling of (**A)** Caspase 3, (**B)** caspase 9, (**C)** VEGF, (**D)** VEGF-R.

The study found that Ha-PLGA-NPs can trigger apoptosis in MCF-7 cells by increasing the expression of caspase 3 and 9 genes (Fig. 7 A, B). The results also showed that the compound has a significant impact on the expression of VEGF and VEGF-R genes (Fig. 7 C, D), suggesting that it may have the potential to inhibit angiogenesis. These findings highlight the potential of Ha-PLGA-NPs as a therapeutic agent for cancer treatment by inducing programmed cell death and inhibiting the formation of new blood vessels. A study by Chen et al. [37] investigated the effects of curcumin-loaded PLGA nanoparticles on breast cancer cells. They reported induction of apoptosis through caspase activation, similar to our findings with harmine-loaded PLGA nanoparticles. In contrast, a study by Singh et al. [38] examined the effects of epigallocatechin-3-gallate (EGCG) loaded SLNs on cancer cells. They demonstrated an increase in caspase 3 expression and a decrease in VEGF expression, indicating apoptosis induction and potential anti-angiogenic activity, respectively. These results align with our findings regarding harmine-loaded PLGA nanoparticles.

### 3.6 Toxicity analysis

The data presented in Table 3 showcases the average daily weight gain and daily food intake for each treatment. Notably, there were significant differences observed between the groups regarding their average daily weight gain and daily food intake, with a p-value of less than 0.05. It was found that increasing the concentration of Ha-PLGA-NPs up to 50 and 100 mg/kg BW resulted in a significant improvement in the average daily weight gain and food intake when compared to the control group. However, it is worth noting that the application of HPF above 100 mg/kg BW demonstrated significant detrimental effects on both weight gain and food intake.

**Table 3:** The body weight and feed intake alteration during the experiment receiving different treatments.

Average	T1	T2	T3	SEM
Average daily weight gain (mg/day)	18.97^c^	42.77^b^	59.62^a^	3.67
Average daily feed intake (g)	1.9^c^	2.8^b^	3.4^a^	0.13

T1: Control.

T2: Mice receiving 50 mg/kg/BW of Ha-PLGA-NPs.

T3: Mice receiving 100 mg/Kg/BW of Ha-PLGA-NPs.

Different letters in the same raw indicated significant differences (p < 0.05).

The analysis was performed in triplicates.

The liver enzyme levels, including AST, ALT, and ALP, alongside antioxidant enzyme levels such as GPx, CAT, and SOD, are crucial indicators for assessing liver damage and toxicity. According to Table 4, the administration of Ha-PLGA-NPs at concentrations of 50 and 100 mg/kg/BW resulted in a significant improvement in these parameters (p < 0.05). Furthermore, the levels of immunoglobulins also showed a similar increase to the liver and antioxidant enzyme levels in mice who received 50 and 100 mg/kg/BW of Ha-PLGA-NPs, suggesting that the nanoparticles assisted in maintaining a normal immune response (Table 4). A study by Zhang et al. [39] explored the effects of curcumin-loaded PLGA nanoparticles on mice body weight and liver function. They reported an increase in body weight and alterations in liver enzyme levels, similar to our findings with harmine-loaded PLGA nanoparticles. On the other hand, a study by Chen et al. [40] investigated the administration of resveratrol-loaded solid lipid nanoparticles (SLNs) and their impact on mice body weight and liver function. They reported no significant changes in body weight or liver enzyme levels following treatment with resveratrol-loaded SLNs.

**Table 4:** The blood parameters analysis during the experiment receiving different treatments.

Parameters	T1	T2	T3	SEM
AST (U/L)	273^a^	214^b^	159^c^	2.65
ALT (U/L)	191^a^	184^b^	143^c^	3.81
ALP (U/L)	404^a^	364^b^	309^c^	5.24
IgA (mg/dl)	4.8^a^	5.5^a^	5.9^a^	0.67
IgG (mg/dl)	5.4^a^	6.2^a^	6.8^a^	0.32
IgM (mg/dl)	6.7^a^	7.1^b^	6.9^a^	0.29
GPX (U/ml)	435^c^	525^b^	740^a^	5.46
SOD (U/ml)	52^b^	60^a^	66^a^	1.44
CAT (U/ml)	30^b^	36^a^	39^a^	2.13

T1: Control.

T2: Mice receiving 50 mg/kg/BW of Ha-PLGA-NPs.

T3: Mice receiving 100 mg/Kg/BW of Ha-PLGA-NPs.

Different letters in the same raw indicated significant differences (p < 0.05).

The analysis was performed in triplicates.

**Fig. 8:**
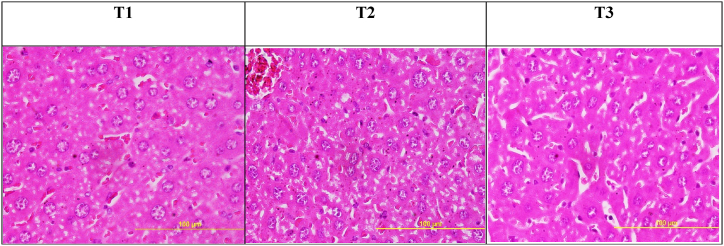
The histopathological assessment in the mice's liver received different treatments.

Upon conducting the histopathological assessment, it was observed that the administration of 50 and 100 mg/kg/BW of Ha-PLGA-NPs, in comparison to the control group, did not exhibit any significant alterations that would provide evidence supporting the claim that harmine-loaded PLGA is non-toxic (Fig. 8).

**Fig. 9:**
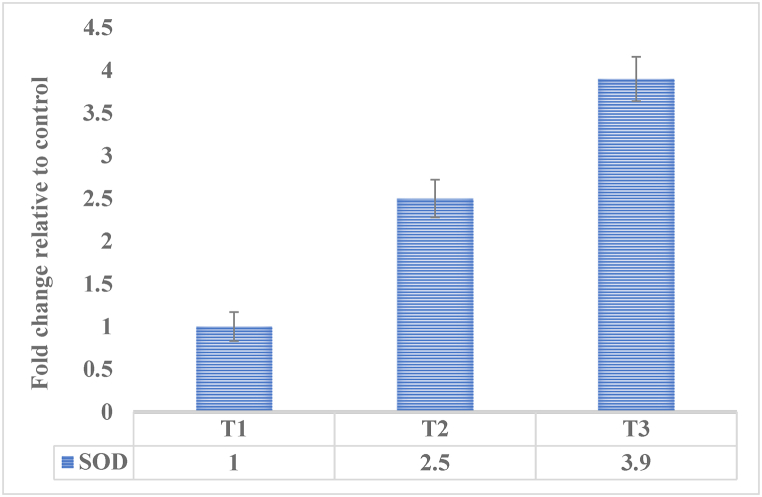
SOD gene expression pattern in the liver of mice received different concentrations of Ha-PLGA-NPs. T1: Control; T2: Mice receiving 50 mg/kg/BW of Ha-PLGA-NPs; T3: Mice receiving 100 mg/Kg/BW of Ha-PLGA-NPs.

The SOD is a key enzyme involved in the elimination of superoxide radicals and plays a crucial role in maintaining the cellular redox balance and protecting against oxidative stress. The increased expression of the SOD gene indicates an upregulation of the antioxidant machinery, which can contribute to the reduction of oxidative damage and maintenance of liver health [41]. As presented in Fig. 9 the SOD expression profile indicates a significant increase in antioxidant enzymes in the mice group receiving 50 and 100 mg/kg/BW of Ha-PLGA-NPs respectively. This increase in antioxidant enzymes demonstrates the cytoprotective role of SOD in normal liver cells. By scavenging harmful free radicals, SOD helps protect liver cells from oxidative damage and maintains their normal function [42,43]. The upregulation of the SOD gene suggests that harmine-loaded PLGA nanoparticles may have the potential to activate the antioxidant defense mechanisms in the liver.

## 4 Conclusion

The synthesis of harmine encapsulated in PLGA nanoparticles represents a significant advancement in the field of cancer therapy. The Ha-PLGA-NPs exhibit potent anticancer and anti-angiogenic activities, along with favorable biocompatibility and safety profiles. Further research efforts should focus on elucidating the underlying mechanisms, optimizing delivery strategies, and conducting comprehensive preclinical studies to pave the way for their translation into clinical applications as efficient anticancer and anti-angiogenic agents.

## Ethics statement

Ethics approval has been obtained by the University Ethics Committee with letter no. IR.IAU.MSHD.REC.1401.097. All methods are reported by ARRIVE guidelines.

## Funding

There has been no financial support for this work.

## Availability of data and materials

The datasets applied during the current study are available upon reasonable request.

## CRediT authorship contribution statement

**Faezeh Mohammadi:** Writing – original draft, Methodology, Investigation, Formal analysis. **Negar Ghaleh navi:** Writing – original draft, Methodology, Formal analysis. **Ehsan Karimi:** Writing – review & editing, Validation, Supervision, Project administration, Formal analysis, Data curation, Conceptualization. **Masoud Homayouni-Tabrizi:** Writing – review & editing, Validation, Supervision, Project administration. **Ehsan Oskoueian:** Writing – review & editing, Methodology, Formal analysis.

## Declaration of competing interest

The authors declare that they have no known competing financial interests or personal relationships that could have appeared to influence the work reported in this paper.
